# Embedding assessment of liver fibrosis into routine diabetic review in primary care

**DOI:** 10.1016/j.jhepr.2021.100293

**Published:** 2021-04-22

**Authors:** Dina Mansour, Allison Grapes, Marc Herscovitz, Paul Cassidy, Jonathan Vernazza, Andrea Broad, Quentin M. Anstee, Stuart McPherson

**Affiliations:** 1 Queen Elizabeth Hospital, Gateshead, UK; 2 Translational & Clinical Research Institute, Faculty of Medical Sciences, Newcastle University, Newcastle upon Tyne, UK; 3 Bewick Road Surgery, Gateshead, UK; 4 Teams Medical Centre, Gateshead, UK; 5 Raigmore Hospital, Inverness, UK; 6 Liver Unit, Newcastle Upon Tyne Hospitals NHS Trust, Freeman Hospital, Newcastle upon Tyne, UK

**Keywords:** Non-alcoholic fatty liver disease, Fibrosis, Liver stiffness, Non-invasive, Serum biomarkers, Transient elastography, Type 2 diabetes mellitus, ALP, alkaline phosphatase, ALT, alanine aminotransferase, AST, aspartate aminotransferase, EASL, European Association for the Study of the Liver, ELF, enhanced liver fibrosis test, GGT, gamma-glutamyl transferase, HbA1c, glycated haemoglobin, HCC, hepatocellular carcinoma, LFTs, liver function tests, LSM, liver stiffness measurement, NAFLD, non-alcoholic fatty liver disease, NASH, non-alcoholic steatohepatitis, OR, odds ratio, T2DM, type 2 diabetes, TE, transient elastography

## Abstract

**Background & Aims:**

Individuals with type 2 diabetes (T2DM) are at high risk of developing non-alcoholic fatty liver disease (NAFLD) and advanced fibrosis/cirrhosis. Screening patients with T2DM and normal liver enzymes for NAFLD in primary care remains contentious. Our aim was to develop and assess a primary care pathway integrating two-tier (Fib-4 then transient elastography [TE]) liver fibrosis assessment, irrespective of aetiology, into routine annual review of all patients with T2DM.

**Methods:**

All patients aged >35 years with T2DM attending annual review at 2 primary care practices in North East England between April 2018 and September 2019 (n = 467) had Fib-4 requested via the electronic patient record. Those with a Fib-4 score above the ‘high-sensitivity’ threshold (>1.3 for ≤65 years and >2.0 for >65 years) underwent TE and were reviewed in secondary care if the liver stiffness measurement (LSM) was >8 kPa. The number of patients identified with advanced disease, service uptake, and predictors of advanced disease were assessed.

**Results:**

A total of 85/467 (18.5%) patients had raised Fib-4; 27/467(5.8%) were excluded as a result of frailty or known cirrhosis. A total of 58/467 (12.2%) were referred for TE. Twenty-five of 58 (43.1%) had an LSM of >8 kPa and 13/58 (22.4%) had an LSM >15 kPa; 4/58 (6.7%) did not attend and 5/58 (9.3%) had an invalid reading. Twenty of 440 (4.5%) patients were found to have advanced liver disease following specialist review, compared to 3 patients previously identified through standard care (odds ratio [OR] 6.71 [2.0–22.7] *p* = 0.0022). Alcohol (OR 1.05 [1.02–1.08] *p* = 0.001) and BMI (OR 1.09 [1.01–1.17] *p* = 0.021) were predictors of advanced disease, particularly drinking >14/21 units/week (*p* <0.0001)

**Conclusions:**

Incorporating 2-tier assessment of liver fibrosis into routine annual diabetes review in primary care significantly improves identification of advanced liver disease in patients with T2DM.

**Lay summary:**

People with type 2 diabetes are at increased risk of developing non-alcoholic fatty liver disease and developing more significant complications. This study looks at introducing screening for advanced liver disease into the annual diabetes reviews performed routinely in primary care; we found that significantly more people were identified as having significant liver disease through this pathway than with current standard care.

## Highlights

• People with type 2 diabetes are at increased risk of advanced liver fibrosis, exacerbated by excess alcohol consumption.
• Incorporating a 2-step fibrosis assessment into primary care diabetes review increased advanced fibrosis detection 7-fold.
• Fib-4 scores automatically calculated at diabetic review in primary care and results displayed with advisory comments.
• Almost half the patients identified with advanced liver disease had alanine transferase below the laboratory cut-off.
• This pathway could improve detection of advanced liver disease in individuals with type 2 diabetes in primary care.

## Graphical abstract

**Figure:**
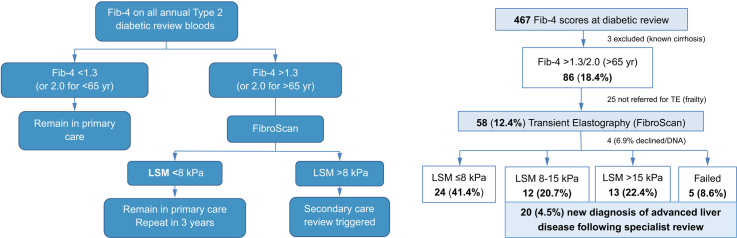

## Introduction

Non-alcoholic fatty liver disease (NAFLD) is the most common cause of liver disease in the Western world.^1^ with an estimated prevalence of 25–30% in the adult population.^2^ Individuals with type 2 diabetes (T2DM) are at higher risk of developing NAFLD, and a greater proportion develop non-alcoholic steatohepatitis (NASH) and progress to fibrosis and cirrhosis.^3^^,^^4^ We face a global epidemic of obesity and T2DM, affecting progressively younger people.^5^ As a result, the prevalence of advanced liver disease attributable to NAFLD is projected to rise exponentially over the coming decade.^6^ Liver disease is generally asymptomatic until the later stages of disease. Currently, in the majority of cases, a diagnosis of NAFLD is made on the basis of abnormal liver blood tests in overweight/obese individuals, or as an incidental finding of fatty liver on imaging, following exclusion of other liver diseases.^7^ Primary care clinicians play a crucial role in the early identification, investigation, and referral of patients with liver disease, but implementing abnormal liver blood test guidelines is associated with a number of challenges: there is a large pool of individuals with risk factors for developing liver disease but only a small proportion will develop clinically significant disease; general practitioners have limited time and resources to investigate patients with abnormal liver blood tests, and there is a lack of access to investigations such as transient elastography (TE) or the enhanced liver fibrosis test (ELF) in primary care.

Even when abnormal liver blood tests are appropriately investigated, significant liver disease can be missed because over 50% of patients with advanced fatty liver disease have normal liver enzymes^8^ and ultrasound has low sensitivity for mild (<20–30%) steatosis and fibrosis.^9^ Consequently, a large proportion of patients present late with decompensated cirrhosis or hepatocellular carcinoma. The North East of England, a ‘hot spot’ for liver disease, exemplifies this; here, preventable mortality in those under 75 years old from liver disease is 22.6 per 100,000 (40% above the national average) and only 11.4% (national average of 15.7%) of patients with hepatocellular carcinoma are treated with curative intent, as a result of late diagnosis.^10^ Earlier recognition of liver disease could potentially alter these outcomes.^11^

However, even in higher risk individuals with T2DM, only a minority of those with NAFLD develop complications of liver disease. Consequently, screening for NAFLD remains contentious, with conflicting guidance from specialist societies. The European Associations for the Study of the Liver (EASL), Diabetes, and Obesity advocate risk-based case finding for NAFLD using abdominal ultrasound in individuals with obesity, T2DM, metabolic syndrome, or incidentally discovered abnormal aminotransferase concentrations.^12^ In contrast, the American Association for the Study of Liver Disease (AASLD) and the National Institute for Clinical Excellence (NICE) in the UK, do not recommend screening for NAFLD in T2DM, citing a lack of reliable tests, the high prevalence with benign course in the majority of patients, and lack of effective pharmacotherapy.^13^^,^^14^

Although there is controversy over the reliability of diagnostic tests for fatty liver, there is widespread agreement in international guidelines on the use of non-invasive tests to identify advanced fibrosis in patients with NAFLD, including the Fib-4 score, NAFLD fibrosis score, TE, and the ELF, all of which have been validated in NAFLD in secondary/tertiary care settings.[15], [16], [17], [18] These tests could be extended beyond patients with a formal diagnosis of NAFLD, to individuals at high risk of developing advanced liver disease, irrespective of aetiology,^19^ although care needs to be taken when applying them to lower disease prevalence settings such as community or primary care. As with many forms of liver disease, liver fibrosis stage is the key prognostic factor in NAFLD, and advanced fibrosis (F3/F4) is associated with a sharp increase in risk of liver-related mortality and all-cause mortality.[20], [21], [22] Identifying these patients is critical, to enable them to access treatment and/or surveillance for manageable complications. Testing for advanced fibrosis (as opposed to fatty liver) would allow simplification of primary care pathways and ensure that the highest risk patients are identified from the large pool of individuals with risk factors for liver disease. It would also identify patients with combined alcohol use disorders and metabolic risk factors, who develop dual aetiology fatty liver disease, which is associated with accelerated progression of liver fibrosis.^23^

A clinical pathway involving a 2-tier staging approach (Fib-4 score followed by ELF or TE) for those with abnormal liver blood tests has been shown to increase the number of patients identified with advanced fibrosis/cirrhosis while reducing the number of referrals for patients with no/mild fibrosis.^24^ In addition, a study from the USA demonstrated that a staged risk stratification using Fib-4 and TE in patients referred from primary care with abnormal liver function tests and/or steatosis on imaging, could save up to 87% of further assessments.^25^ Given that patients with T2DM already have regular clinical reviews including liver blood tests, there is an opportunity to incorporate ‘liver fibrosis assessment’ to improve detection of advanced liver disease in patients with diabetes.^15^ The aim of this pilot study was to assess the impact of implementing a 2-tier staging pathway (Fib-4 score followed by TE) in patients undergoing routine diabetes review in primary care.

## Materials and methods

The pathway to identify significant liver fibrosis in patients with T2DM was developed as a service innovation in collaboration with General Practitioners and the Newcastle and Gateshead Primary Care Clinical Commissioning Group, and run as a pilot in 2 GP practices in Gateshead between April 2018 and September 2019. The working group met regularly to develop the pathway before implementation. Changes were made to the shared electronic health record system (Sunquest ICE) to allow Fib-4 to be requested from primary care, avoiding the need to calculate the score by hand. ‘Pop-up’ alerts were also set up on ICE to guide GPs through the pathway and prompt TE request or referral as appropriate. Patients and the public were involved in the development of the pathway through local patient group (diabetes.uk and LIVErNORTH) meetings.

This was carried out as a service development project. Therefore, this study was registered for audit (audit number NG049) but not subject to review by an independent ethics committee, and written consent was not sought. All activities were performed in accordance with the guidelines of the Helsinki Declaration.

### The pathway

**Fig. 1:**
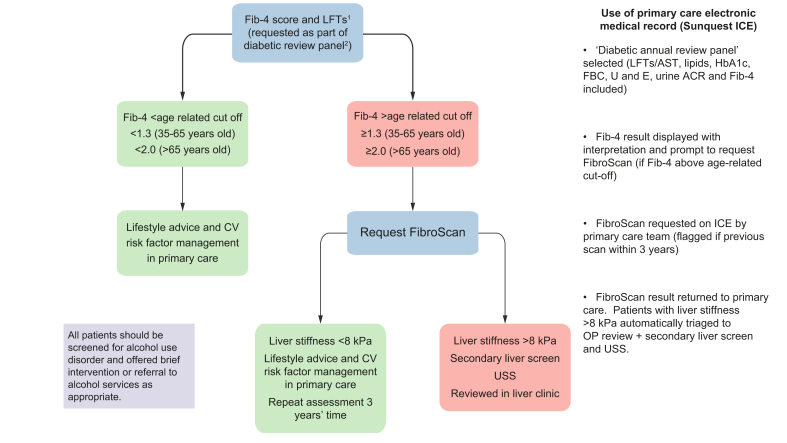
The pathway identifying significant liver disease in patients with type 2 diabetes. All patients with abnormal ALT/ALP also had liver aetiology screen as per national and regional guidelines on investigation of abnormal liver blood tests. Right hand box explains how modifications to the patient electronic medical record lead the primary care teams through the pathway. ACR, albumin-creatinine ratio; AST, aspartate aminotransferase, CV, cardiovascular; FBC, full blood count; HbA1C, glycated haemoglobin; LFT, liver function tests (comprising: albumin; ALP, alkaline phosphatase; ALT, alanine aminotransferase; bilirubin; GGT, gamma-glutamyl transferase); OP, outpatient; U and E, urea and electrolytes.

The pathway is summarised in Fig. 1. All patients aged >35 years old with T2DM undergoing annual review in the 2 participating practices had a Fib-4 requested within the electronic patient record in addition to routine blood tests. The system automatically reported the Fib-4 score result without the need to calculate the score manually. Patients under 35 years old were referred only if there was biochemical or radiological evidence of liver disease, as Fib-4 does not perform well in patients under 35 years old.^18^ Patients were given information on the pilot pathway, and verbal consent was gained to participate. Patients with a Fib-4 score above the age-related high-specificity cut-off (>1.3 in patients ≤65 or >2.0 in patients >65),^18^^,^^26^ were considered at increased risk of advanced fibrosis, and were offered TE (FibroScan). TE was not offered to patients with severe frailty (assessed using the electronic Frailty Index, or equivalent) or life-limiting conditions, where the referring primary care team felt that further investigations were not in the patient’s best interests. Patients with a Fib-4 score below the age-related cut-off were managed in primary care.

Patients were either scanned at their primary care centre or at the local hospital. Those who did not attend their initial appointment were telephoned, asked for the reason for their non-attendance, and given a second appointment.

TE was performed by 2 experienced specialist liver nurses using the portable FibroScan FS402 device (Echosens, Paris, France) as previously described.^27^ Patients with a BMI of greater than 35 kg/m^2^ underwent TE using the XL probe (FS502 device), owing to the high rate of scan failures using the standard M probe above this BMI threshold.^28^ The liver stiffness measurement (LSM) represented the median value of successful measurements, measured in kilopascals. A scan failure was defined as the inability to obtain 10 valid TE measurements in an individual or if the IQR/median was greater than 0.3 as per recent guidance.^29^

Patients who had an LSM <8 kPa were considered to be low probability of having advanced liver fibrosis and remained in primary care, to have repeat assessment in 3 years. Patients with an LSM ≥8 kPa, indicative of significant hepatic fibrosis,^30^ were reviewed in secondary care and had a liver aetiology screen and ultrasonography performed, where appropriate. Following review in specialist clinics, further investigations, including liver biopsy, were arranged at the discretion of the reviewing clinician and/or patients were enrolled into cirrhosis surveillance programmes. All patients attending for TE were offered verbal and written lifestyle advice. Those who also had a history of high-risk alcohol use were referred to local alcohol services.

### Analysis

Statistical analysis was performed using SPSS version 24 (IBM, Armonk, NY, USA).

The primary outcome of this real-world study was the number of patients with advanced fibrosis/cirrhosis identified through the pathway. Secondary outcomes were service uptake (number of patients declining or not attending tests), and predictors of advanced fibrosis. To examine the potential additional benefit the Gateshead pathway over other pathways that only investigate patients with raised liver enzymes, we also compared the number of patients identified with advanced fibrosis/cirrhosis through the Gateshead pathway *vs.* those identified if only those with raised liver enzymes were investigated as per British Society of Gastroenterology guidance.^14^

Diagnosis of ‘advanced liver disease’ was made on specialist review, using the following established criteria:

1) Radiological evidence of cirrhosis (splenomegaly, nodular liver, ascites or varices).
2) Oesophageal or gastric varices on endoscopy.
3) F3 or F4 fibrosis on liver histology, following review in specialist clinics.
4) Diagnosis of cirrhosis based on overall clinical assessment.

A liver stiffness >15 kPa on FibroScan alone was not used to diagnose advanced liver disease; 2 recent multicentre studies in NAFLD^16^^,^^31^ have shown that using liver stiffness as a surrogate of advanced fibrosis results in a high number of false-positive results (30–40%) as compared with liver biopsy. Patients with LSM >15 kPa but no imaging or endoscopic evidence of portal hypertension were offered liver biopsy to make a definitive diagnosis.

Patient BMI, alcohol intake, and features of the metabolic syndrome were taken from primary and/or secondary care records – where BMI/alcohol intake was not available this was recorded as missing data. Descriptive data are presented for TE attendance, stratification of liver disease (based on LSM and subsequent diagnosis of ‘advanced liver disease’). Categorical data are presented as numbers (percentage). Continuous data are presented as mean (standard deviation) for normally distributed data and median (range) for non-normal data.

Univariate analysis to compare the characteristics of participants with/without ‘advanced liver disease’ was undertaken using Student’s *t* test (normal continuous), Mann-Whitney *U* test (non-normal continuous) and the χ^2^ test (categorical). Multivariate logistic regression analysis of age, alcohol intake, BMI, and sex was used to define independent predictors of advanced liver disease.

## Results

A total of 475 consecutive patients with T2DM (all patients with T2DM registered at both practices) attended routine annual diabetes review during the period of the study.

Overall, 466/475 patients were aged over 35 years and had a Fib-4 score requested. A summary of their patient characteristics is shown in Table 1. Three were known to have cirrhosis and were excluded from analysis. Eighty-two patients (17.7%) had a Fib-4 score above the age-related cut-off (≥1.3 for aged ≤65 and under, ≥2.0 for >65 years old) and 55 (67% of those with raised Fib-4 or 11.9% of those with Fib-4 tested) were referred for TE. Of the remaining 24 who were not referred for FibroScan, 20 were considered unsuitable for further investigation by the referring GP because of frailty/life limiting illness (17), inability to give consent (1), or were already known to gastro/hepatology services (2), and 4 patients died during the pilot period (cause of death recorded as aortic abscess, heart failure and COPD, pancreatitis and pneumonia).

**Table 1:** **Patient characteristics**.

	All patients∗ (466)	Patients referred for TE∗∗ (58)
Male	293 (61.8%)	34 (69.4%)
Age, years	**63.82 (13)**	**65.22 (9.87)**
35–45	33 (7.1%)	2 (3.4%)
46–55	67 (14.4%)	7 (12.1%)
56–65	137 (29.4%)	24 (41.4%)
66–75	130 (27.9%)	15 (25.9%)
76–85	76 (16.3%)	9 (15.5%)
>85	23 (4.9%)	1 (1.7%)
Alcohol (units/wk†)	**5.0 (0**–**90)**	**9 (0**–**90)**
<14/21†	401 (84.6%)	45 (77.6%)
14–35/21–50†	29 (6.2%)	9 (15.5%)
>35/50†	8 (1.6%)	2 (3.4%)
Missing data	36 (7.6%)	2 (3.4%)
BMI	**32.62 (6.38)**	**33.36 (7.27)**
<25	41(9.4%)	4 (6.9%)
25–30	146 (33.3%)	19 (32.8%)
>30	251 (57.3%)	20 (34.5%)
Missing data	36 (7.6%)	4 (6.9%)
HbA1c	**35–139**	**42–123**
Median (IQR)	**66 (14)**	**56 (25)**
On statin (%)	88.6%	86.6%
Hypertensive (%)	73.8%	67.3%
Metabolic syndrome (%)	43.5%	53.8%
Platelet count	**252.4 (69.0)**	**182.2 (52.2)**
GGT	**59.0 (88.0)**	**92.4 (100.2)**
ALT	**24.6 (14.29)**	**31.15 (17.5)**

Continuous variables presented as mean ± standard deviation (BMI, plt, GGT, ALT, HbA1c)/ median with range and IQR (alcohol) in bold or as number of participants (%). Metabolic syndrome is defined as having a combination of any 3 of diabetes, obesity (BMI >30), dyslipidaemia, and/or hypertension. ALT, alanine aminotransferase; GGT, gamma-glutamyl transferase; HbA1c, glycated haemoglobin; IQR, interquartile range; plt, platelets; TE, transient elastography.

† Units of alcohol consumed per week in females/males respectively.

∗ All patients >35 years old attending for diabetes annual review (n = 466).

∗∗ Patients referred for Transient Elastography (n = 58).

**Fig. 2:**
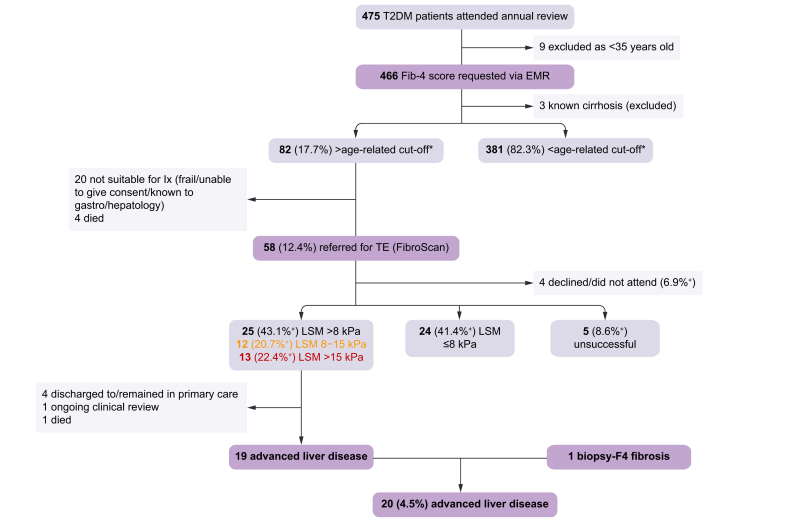
Results of implementation of the pathway. F4 fibrosis equates to cirrhosis as per the SAF (steatosis, activity, fibrosis) score, advanced liver disease was diagnosed in those with liver stiffness >8 kPa who had clinical, endoscopic, imaging, or histological evidence of portal hypertension or advanced liver disease. ∗Age-related cut-off for Fib-4 was ≥1.3 for 35–65-year-olds and ≥2.0 for >65-year-olds. ^†^Expressed as percentage of patients referred for FibroScan. EMR, electronic medical record; Ix, investigations; LSM: liver stiffness measurement; T2DM: type 2 diabetes mellitus, TE: transient elastography.

Of the 58 patients referred for TE, 49 (84.5%) patients had valid LSM results, 5 (9.3%) patients had invalid LSM and 4 (7%) individuals did not attend. The results of the patient assessments are summarised in Fig. 2.

In total, 25 of the 58 patients referred for TE (43.1%; 5% of all patients) had an LSM of >8 kPa suggestive of significant fibrosis (28), and 13 (22.4%; 3%) had an LSM >15 kPa indicative of advanced fibrosis or cirrhosis (29).

23 of the 25 patients with an LSM >8 kPa were reviewed in hepatology clinics, including all 13 of those with LSM >15 kPa. All liver aetiology screens were negative. The outcomes following specialist review and investigation are shown in Table 2. In total 20/23 (87%) of patients referred to the hepatology clinic were found to have advanced liver disease following specialist review: 3/20 (15%) had endoscopic evidence of portal hypertension (gastro-oesophageal varices or portal hypertensive gastropathy); 14/20 (38.5%) had imaging evidence of liver cirrhosis and/or hepatocellular carcinoma and 3/20 (15%) were diagnosed with F3/F4 fibrosis on liver biopsy. These patients were entered into surveillance programmes. Three patients with advanced liver disease were already known to liver services, giving an overall prevalence of advanced liver disease in this population of 4.8%. There was an almost 7-fold increase in the detection of advanced liver disease compared with standard care in place before the pilot (4.55% *vs.* 0.67% odds ratio [OR] 6.71, 95% CI 2.0–22.7 *p* = 0.0022). There were further significant changes in management as a result of the pathway: 2 asymptomatic patients were diagnosed with hepatocellular carcinoma on their initial ultrasound scan (one 56-year-old man with F3 fibrosis was referred for curative resection and 1 with more advanced disease was treated with best supportive care); 2 patients discontinued methotrexate therapy after being found to have cirrhosis, and 2 patients with gastric/oesophageal varices received primary prophylaxis.

**Table 2:** **Outcomes following specialist review**.

Age, yr	M/F	Alcohol (u/wk)	Plt	LSM (kPa)	Imaging	Endoscopy	Biopsy	Advanced disease	Management and outcome (1 year)
43	M	0	81	74.5	Nodular liver, splenomegaly	Grade 2 oesophageal varices	–	Yes	HCC surveillance Variceal band ligation
79	M	65	190	10.1	Steatosis	–	–	–	Discharged to primary care with repeat FibroScan at 1 year
51	M	95	145	16.1	Pancreatitis/fatty liver	–	–	–	Died – pancreatitis and endocarditis
67	M	70	228	9.3	Hepatocellular carcinoma	–	F3 fibrosis	Yes	Referred for curative treatment
76	M	10	142	10.5	Nodular liver	Normal	F3 fibrosis	Yes	HCC surveillance
64	F	0	260	14.8	Steatosis	–	Declined	–	Clinical follow-up
58	M	70	180	30.7	Nodular liver	Declined	Declined	Yes	HCC surveillance
53	M	90	127	15.9	Nodular liver, splenomegaly	–	–	Yes	HCC surveillance
56	F	35	258	27.4	Re-canalised umbilical vein	Normal	–	Yes	HCC surveillance
52	F	16	205	13.4	Nodular liver, splenomegaly	–	–	Yes	HCC surveillance
59	M	0	188	19.4	Irregular fatty liver	–	Declined	Yes	HCC surveillance
64	F	0	159	12.0	Fatty liver, splenomegaly	–	Declined	Yes	HCC surveillance
54	F	2	244	24.5	Splenomegaly	Portal hypertensive gastropathy	–	Yes	HCC surveillance
58	F	0	227	17.3	Steatosis	–	F2 fibrosis	–	9.5-kg weight loss Biopsy F2 fibrosis Discharged to primary care
59	M	0	136	17.1	Fatty liver, splenomegaly	–	–	Yes	HCC surveillance
58	M		258	9.6	Steatosis		–	–	Repeat FibroScan 7.5 kPa – discharged to primary care
64	F			8.6	Normal		–	–	Did not attend clinic
67	M	0	119	10.4	Irregular liver, splenomegaly	–	–	Yes	HCC surveillance
78	M	0	175	13.9	Coarse nodular liver	–	Declined	Yes	HCC surveillance
89	M	0	222	26.6	Hepatocellular carcinoma	–	–	Yes	Best supportive care
67	M	0	313	34.0	Splenomegaly, gastric varices	Gastric fundal varices	–	Yes	HCC surveillance carvedilol Methotrexate stopped
67	M	0	119	12.4	Coarse echotexture, splenomegaly	–	–	Yes	Discharged to primary care (patient choice, frail)
77	M	0	109	22.0	Nodular liver	Declined	Declined	Yes	HCC surveillance, methotrexate stopped
50	M	0	180	13.8	Splenomegaly, coarse liver	–	Declined	Yes	HCC surveillance
81	M	0	110	44.1	Irregular liver, splenomegaly	Normal	–	Yes	HCC surveillance

Table showing age, sex, alcohol intake, platelet count, liver stiffness measurement, results of imaging, endoscopy (Oesophago-gastro-duodenoscopy), liver biopsy (if applicable) of each patient referred to secondary care through the pathway, alongside whether they were identified as having advanced disease, and their management and outcome at the end of the pilot period (up to 1 year after initial assessment). F, female; HCC, hepatocellular carcinoma; LSM, liver stiffness measurement; M, male; plt, platelets; u/wk, units per week.

### Service uptake

In total 7% patients (4/58) referred for TE did not attend or declined the test after 2 offers of an appointment. 10 of 58 (17%) did not attend or declined the initial appointment but attended after a second invitation. We found that patients were more likely to attend for TE at their local GP surgery than a hospital clinic. None of the patients offered the scan at their GP surgery failed to attend, although 2/35 (6%) declined the test. In contrast, the initial non-attendance rate at the hospital clinic was up to 75% (12/16). When patients were asked why they declined or did not attend the test, 4/14 (29%) patients did not feel they needed the test – “I don’t have a problem with my liver – my doctor always says my liver tests are fine”; 3/14 (21%) thought the test was voluntary and for research or interest only; 4/14 (29%) said that the test was at an inconvenient time or they were unable to take time off work, and 3 (21%) said they found it difficult to get to or park their car at the hospital.

### Impact of this screening pathway on the detection of advanced liver disease compared with investigating abnormal liver blood tests

Current UK national guidelines do not recommend screening for NAFLD in high-risk groups and as a result most patients with NAFLD are diagnosed following investigation of abnormal liver blood tests. The majority of laboratories analyse only alanine aminotransferase (ALT) unless aspartate aminotransferase (AST) and gamma-glutamyl transferase (GGT) are specifically requested. Overall, 45.5% of patients with advanced disease in this study had a normal ALT (see Table 3), and so would have been missed if only the presence abnormal ALT was used to identify liver disease. Using the lower ALT cut-off of <20 for females and <30 for males improved sensitivity of ALT (from 54.4% to 70.0%, but reduced specificity (from 90.2% to 69.1%).

**Table 3:** **Liver blood tests by disease stage**.

Liver blood tests	TE liver stiffness measurement (kPa)			Advanced disease			
	<8 n = 24	8–15 n = 12	>15 n = 13	Yes	No	Sens	Spec
ALT (med & IQR)	20 (9)	41 (21.5)	31 (28)	40 (14.25)	21 (14)		
n (%) ALT>40	1 (4.2%)	7 (58.3%)	6 (46.2%)			54.5%	90.2%
n (%) ALT>20 (women)/>30 (men)	5 (20.3%)	7 (58.3%)	7 (53.8%)			70.0%	69.1%
AST (med & IQR)	25 (9.75)	37 (25.25)	40 (30)	38 (29.25)	19 (8)		
n (%) AST>40	2 (8.3%)	5 (41.7%)	8 (61.5%)			59.1%	90.2%
GGT (med & IQR)	34 (22.5)	64 (117.5)	95 (92)	79 (99)	35 (33)		
n (%) GGT>45	7 (29.2%)	8 (66.6%)	12 (92.3%)			86.4%	54.5%
Plt (med & IQR)	167 (54.5)	213 (65)	164 (52)	184 (84.5)	259 (85)		
n (%) plt<150	5 (20.8%)	3 (25.0%)	5 (38.5%)			33.3%	98.0%

Liver blood tests in individuals with liver stiffness measurement <8 kPa, 8–15 kPa, and >15 kPa, and with and without advanced disease, expressed as median (IQR), number (%) of patients with values outside normal range, and sensitivity and specificity of blood test cut-offs for advanced disease. ALT, alanine aminotransferase; AST, aspartate aminotransferase; GGT, gamma-glutamyl transferase; IQR, interquartile range; plt, platelet count; Sens, sensitivity; Spec, specificity; TE, transient elastography.

### Predictors of advanced disease

The proportion of patients recorded as hazardous alcohol use (>35 units/week for a woman or >50 units/week for a man) on the General Practice systems was low (1.6%). Overall, 84.6% of the whole cohort, and 76.6% of patients referred for TE, were documented as drinking less than 14 (females)/21 (males) units of alcohol/week. Despite this, we found that alcohol intake was a significant predictor of advanced disease, and the prevalence of advanced liver disease was significantly higher in patients drinking more than 14/21 units alcohol/week (31.0% *vs.* 4.4% *p* <0.0001).

BMI and male sex were also significantly associated with advanced disease on univariate analysis (Table 4**)**. Interestingly, increasing age was not significantly associated with advanced disease. This may be as a result of the use of age-related Fib-4 cut-offs, combined with the exclusion of elderly frail patients from further investigation, but may also reflect patients with T2DM and NAFLD developing advanced disease at a younger age.

**Table 4:** **Relationship between age, alcohol intake, BMI, and sex and the presence/absence of advanced liver disease**.

	Advanced liver disease	No advanced liver disease	Wald	Sig	Exp(B)	95% CI for Exp(B) Lower Upper	
Age, years, mean (SD)	64.82 (11.58)	63.35 (12.23)	0.205	0.651	1.01	0.97	1.06
Alcohol units/week mean (SD)	11.67 (20.6)	4.40 (8.1)	10.534	0.001∗	1.05	1.02	1.08
BMI, mean (SD)	36.50 (7.2)	32.26 (6.5)	5.310	0.021∗	1.09	1.01	1.17
Sex, % male	72.7	60	0.849	0.357	0.57	0.17	1.89

This table shows the mean age, alcohol intake (units/week), and BMI in individuals with and without advanced liver disease, and multivariable logistic regression analysis using age, alcohol, and BMI as continuous independent variables, sex as a categorical independent variable, and advanced disease (yes/no) as categorical dependent variable. Wald, significance (sig) and odds ratio for each unit increase in age/BMI/alcohol units and for male sex [Exp(B)] including 95% CIs for Exp(B) shown.

∗ Significance level *p* = 0.05.

On multivariate logistic regression analysis, increasing BMI and alcohol remained independent predictors of advanced disease (BMI: OR 1.09 [1.01–1.17], *p* = 0.021; alcohol: OR 1.05 [1.02–1.08], *p* = 0.001), although BMI over 30 did not reach statistical significance in predicting advanced disease (χ^2^ 2.267, *p* = 0.132). Age and sex were not predictors of advanced disease on multivariate analysis (age *p* = 0.651, sex *p* = 0.357).

## Discussion

In this study we effectively embedded a liver fibrosis assessment into primary care diabetes clinics, and we were able to screen all patients for significant liver disease as part of their annual diabetes reviews. Developments in the electronic medical record system to support the pathway ensured good compliance in primary care, and a high rate of appropriate referrals into secondary care. As a result of the pathway, 20 new cases of advanced liver disease were identified from a cohort of 475 patients, representing almost a 7-fold increase from standard care in this cohort of higher risk patients with T2DM diabetes, including patients with dual aetiology (alcohol and metabolic) fatty liver disease. Three patients were already known to have liver cirrhosis, giving an overall prevalence of advanced disease of 4.8%.

This is in line with a French study by Jacqueminet *et al.*,^32^ where the prevalence of confirmed advanced fibrosis was 4.3% in a population with T2DM who were aged 45 years or older. This prevalence rate is slightly lower than in some other recent studies looking at advanced fibrosis in patients with T2DM; for example, in a recent study from the USA, 9.2% of patients with T2DM were found to have F3/F4 fibrosis.^33^ However, these studies rely mainly on TE to determine advanced fibrosis – in our study patients were only classed as having advanced disease if they had biopsy evidence of F3/F4 fibrosis or endoscopic or imaging evidence of portal hypertension or hepatocellular carcinoma. It should also be noted that, in contrast to most NAFLD studies, our pathway did not exclude patients with a history of potentially harmful alcohol consumption – therefore we would expect a higher prevalence of advanced liver disease.

Almost half of patients subsequently diagnosed with advanced liver disease had a normal ALT level and would have been missed if only liver enzymes were used to identify liver disease. Conversely, only 1 in 5 patients with an abnormal ALT level were identified as having advanced liver disease. Using the lower ALT cut-off of <20 for females and <30 for males would reduce the number of patients with advanced disease missed to 1 in 3, but risks increasing the number of unnecessary referrals. Therefore, our data indicate that using abnormal liver blood tests alone are an ineffective way of detecting advanced liver disease, whereas a 2-stage pathway using Fib-4 score and TE more accurately identified patients with advanced liver disease.

Our pathway provides a simple, standardised approach to assessing liver fibrosis in all patients with T2DM, irrespective of alcohol intake. There is no requirement for pre-screening, and primary care teams are supported by prompts via patients’ electronic medical records. This means that the pathway can be embedded into chronic disease monitoring and implemented by practice nurses and healthcare assistants, thus minimising strain on general practitioners. It also streamlines the patient journey, reducing the number of patient attendances to primary care and unnecessary referrals to secondary care. Measuring Fib-4 on an annual basis as part of diabetic review may also improve detection of significant liver fibrosis, compared with repeating Fib-4 every 3 years, as a recent study has suggested that serial measurement of the Fib-4 score may help identify patients at risk of advanced fibrosis.^34^

There was a high diagnostic yield of significant liver disease amongst patients undergoing TE: over half with a valid reading (25/49) had a liver stiffness >8 kPa, and over one-third of patients scanned (20/54) were found to have advanced disease on subsequent review. A larger number of patients than would be expected with LSM >8 kPa (20/25) had evidence of portal hypertension on imaging/endoscopy, or hepatocellular carcinoma. The reason for this is not clear. It may be because of a relatively high overlap with high alcohol intake (5/20 had alcohol intake >35 units/week), which increases the pre-test probability of advanced fibrosis – however, the numbers are too small to draw any firm conclusions.

The initial screening test (Fib-4) in the pathway is cost free, as the component blood tests (full blood count and liver enzymes) are generally part of the annual diabetic review blood panel in primary care. When the Fib-4 request is added to the diabetic review blood panel, the calculation is automated through the laboratory software and the result displayed, along with an advisory comment, in the patient’s electronic record – this minimises the time required to implement the pathway in primary care. This makes it a highly cost-effective way of improving detection of advanced liver fibrosis. Crucially, a number of patients identified through the pathway had a significant change in their management as a result of a timely diagnosis of advanced liver disease, including diagnosis of hepatocellular carcinoma (with 1 patient undergoing curative treatment), primary prophylaxis for gastro-oesophageal varices and cessation of methotrexate treatment.

Overall, the number of patients who did not attend their appointments for TE was low (7%), although 17% missed their first appointment but attended a second after a phone call to explain the rationale of the test. This emphasises the importance of ensuring that patients are given an adequate explanation of the pathway including the significance of raised fibrosis tests and the need for TE. Patients were more likely to attend for TE when this was performed at their local GP surgery, indicating that uptake of the pathway could be optimised by offering TE in primary care.

BMI and alcohol intake were independent predictors of advanced disease. The prevalence of advanced disease in patients drinking more than 14/21 units of alcohol/week was significantly higher than in patients drinking less than 14/21 units/week (33% *vs.* 4.4% *p* <0.0001), despite only a small number of patients (1.6%) reporting ‘high risk’ alcohol use (>35/50 units/week). This once again demonstrates the importance of alcohol intake as a cofactor in the progression of fibrosis in patients with metabolic risk factors for liver disease and emphasises the importance in including these patients with compound risk factors in referral pathways.

### Limitations

As this was a pragmatic real-world study; GPs and secondary care clinicians were able to use their discretion as to which patients were referred for further investigations, and which were offered ongoing surveillance and follow-up. The number of liver biopsies performed was low and, whilst all patients diagnosed with ‘advanced liver disease’ had either histological, imaging, or endoscopic evidence of advanced disease, we were unable to make a definitive fibrosis assessment in 3/25 patients with LSM >8 kPa. The majority of patients offered a liver biopsy declined and opted for clinical follow-up or surveillance. This may change as the pathway becomes established and with the emergence of new treatments, but once again emphasises the importance of developing more reliable non-invasive biomarkers. In addition, as only patients with a Fib-4 score above the age-related cut-offs were referred for TE, we may have missed patients with advanced disease who have a Fib-4 score below the age-related cut-offs. Some studies have suggested that the cut-off for Fib-4 should be lowered if we wish to optimise its negative predictive value,^35^ and others have found that advanced disease can be missed when using the age related cut-off.^24^ More research is needed to establish the optimum cut-offs for Fib-4 in this population.

### Future developments

Our pathway involved a 2-stage assessment with Fib-4 and TE, based on investigations available locally. Using Fib-4 in combination with a blood biomarker, such as ELF, could potentially streamline the pathway, as they can be reflex-tested following a raised Fib-4 score, without the patient being recalled for a second investigation. A 3-tier assessment incorporating sequential Fib-4, ELF, and TE may reduce the need for liver biopsy further. Recent studies have suggested that combining TE with ELF would significantly reduce the number of patients requiring liver biopsy to diagnose ≥F3 fibrosis in hepatitis B.^36^ Further work is required to design the optimum, most cost-effective pathway, combining non-invasive testing to minimise the need for liver biopsy in patients with NAFLD.^15^ Further research is also required into the optimum cut-offs of Fib-4, ELF, and TE in this population.^15^

Finally, to promote a ‘culture change’ in primary care and to manage the increased demand on secondary care services, we also have to consider the downstream pathways, and find more effective ways of managing patients with significant or advanced liver disease once they have been identified. In the absence of any licensed drug treatment, this involves developing a more structured approach to lifestyle intervention, across primary and secondary care, careful risk stratification, and cost-effective monitoring and surveillance. As these patients do not have, and may never develop, clinical features of decompensation, they may not require face-to-face outpatient appointments in secondary care. We should therefore explore alternative ways of monitoring and surveillance including mobile health technology, and consider surveillance in primary care for a select group of patients.

In conclusion this proof-of-concept study suggests that embedding a 2-tier assessment of liver fibrosis into routine annual diabetes review could successfully improve identification of patients with advanced liver disease by providing a systematic, standardised approach and incorporating it into management of chronic disease in primary care.

## Financial support

The authors received no financial support to produce this manuscript.

## Authors’ contributions

Collected data: PC, MH, DM

Performed analysis and wrote the paper: DM with support from SM

Contributed to development of the pathway: all authors

Contributed to the final manuscript: all authors

## Data availability statement

Owing to the nature of this research, participants of this study did not agree for their data to be shared publicly, so supporting data is not available.

## Conflicts of interest

The authors declare no conflicts of interest that pertain to this work.

Please refer to the accompanying ICMJE disclosure forms for further details.
