# Supplementary material for: Embedding assessment of liver fibrosis into routine diabetic review in primary care
Source: JHEP Rep. 2021 Apr 22;3(4):100293. doi: 10.1016/j.jhepr.2021.100293 (PMC8213901; doi:10.1016/j.jhepr.2021.100293)
Supplement: Multimedia component 1 [file mmc1.pdf]

JHEP|Reports

JHEP Reports

CTAT methods

Tables for a “Complete, Transparent, Accurate and Timely account” (CTAT) are now mandatory for all revised submissions. The aim is to enhance the reproducibility of methods.

- Only include the parts relevant to your study
- Refer to the CTAT in the main text as ‘Supplementary CTAT Table’
- Do not add subheadings
- Add as many rows as needed to include all information
- Only include one item per row

If the CTAT form is not relevant to your study, please outline the reasons why:

Real world study using standard care clinical tests only.

1.1 Antibodies

Name	Citation	Supplier	Cat no.	Clone no.

1.2 Cell lines

Name	Citation	Supplier	Cat no.	Passage no.	Authentication test method

1.3 Organisms

Name	Citation	Supplier	Strain	Sex	Age	Overall n number

1.4 Sequence based reagents

Name	Sequence	Supplier

1.5 Biological samples

Description	Source	Identifier

1.6 Deposited data

Name of repository	Identifier	Link

1.7 Software

Software name	Manufacturer	Version

1.8 Other (e.g. drugs, proteins, vectors etc.)

1.9 Please provide the details of the corresponding methods author for the manuscript:

Dina Mansour, Consultant, Queen Elizabeth Hospital, Sheriff Hill, Gateshead NE9 6SX tel +44191 4452367 e-mail Dina.mansour@nhs.net

2.0 Please confirm for randomised controlled trials all versions of the clinical protocol are included in the submission. These will be published online as supplementary information.

